# Differences in secretome in culture media when comparing blastocysts and arrested embryos using multiplex proximity assay

**DOI:** 10.1080/03009734.2018.1490830

**Published:** 2018-10-04

**Authors:** Karin E. Lindgren, Fatma Gülen Yaldir, Julius Hreinsson, Jan Holte, Karin Kårehed, Inger Sundström-Poromaa, Helena Kaihola, Helena Åkerud

**Affiliations:** a Department of Women’s and Children’s Health, Uppsala University, SE-751 85Uppsala, Sweden;; b Department of Immunology, Genetics and Pathology, Uppsala University, SE-751 85Uppsala, Sweden;; c Department of Clinical Sciences, Intervention and Technology, Karolinska Institute and Unit for Reproductive Medicine, Karolinska University Hospital, SE-14186Stockholm, Sweden;; d Carl von Linné Clinic, SE-751 83Uppsala, Sweden

**Keywords:** Blastocyst, caspase-3, extracellular matrix metalloproteinase inducer, interleukin-6, prediction, secretome, time-lapse, vascular endothelial growth factor A

## Abstract

**Objectives:** The aim of this study was to assess different patterns of the human embryo secretome analysed as protein levels in culture media. Furthermore, analyses to correlate protein levels with quality and timing to development of human embryos were performed.

**Material and methods:** Human day-2 cryopreserved embryos were cultured for four days in an EmbryoScope^®^ with a time-lapse camera, and embryo quality was evaluated retrospectively. After culture, the media were collected and relative levels of secreted proteins were analysed using Proseek Multiplex Assays. Protein levels were evaluated in relation to timing to development and the ability to form a blastocyst.

**Results:** Specific patterns of timing of development of blastocysts were found, where a difference in time to start of cavitation was found between high- and low-quality blastocysts. There appeared to be a correlation between specific protein patterns and successful formation of morulae and blastocysts. Embryos developing into blastocysts had higher levels of EMMPRIN than arrested embryos, and levels of caspase-3 were lower in high- versus low-quality blastocysts. Also, higher levels of VEGF-A, IL-6, and EMMPRIN correlated with shorter times to morula formation.

**Conclusions:** The secretome and timing to development differ in embryos forming blastocysts and those that become arrested, and in high- versus low-quality blastocysts. The levels of certain proteins also correlate to specific times to development.

## Introduction

Infertility affects about 15% of all couples of reproductive age. Assisted reproduction through *in vitro* fertilization (IVF) can be used as a treatment for these couples. IVF has been performed routinely for many years, although pregnancy rates have not improved much in recent years, and, according to Calhaz-Jorge and colleagues (1) and the Swedish National Quality Register for Assisted Fertilisation (Q-IVF; www.medscinet.com/qivf), the success rate after IVF treatment in Sweden as of today is approximately 25–30%. Success rate is measured as live born infants after IVF treatment. There is a demand for improved prediction models to increase the implantation rate. Early prediction of the viability of human embryos developed and matured *in vitro* still remains challenging. The predominant non-invasive technique for selecting embryos is based on morphology (2), where variables such as timing of cleavage and blastocyst formation as well as developmental kinetics are evaluated. The morphological approach, however, is inadequate for prediction of embryo quality and success rate after transfer, meaning a live born infant. In recent years several studies have been performed that have been focused on the development of new and more sophisticated non-invasive methods for evaluation of embryo quality (3–5). Among the methods used, different approaches have been investigated, including proteomics, metabolomics, and genomics (6–8). The prediction models used in IVF clinics today are mainly based on general health factors such as body mass index (BMI), age, smoking, and reason for infertility, as well as biological markers and finally morphology of the embryo during development.

Monitoring embryo development by means of a time-lapse technique has improved embryo selection during IVF, although the method is considered subjective and non-standardized (9). In recent years, more quantitative methods and algorithms for embryo selection have been sought, and new algorithms aimed at use in clinical praxis are being evaluated frequently (10–15). Differences in protein expression related to the development and viability of the early embryo have been described by Katz-Jaffe and McReynolds (16). Until day 3, when the human embryo consists of four to six cells, maternal transcripts and proteins support embryo development and differentiation. Zygotic genome activation starts at day 3, whereas most of the maternal transcripts rapidly disappear and are replaced by novel embryonic transcripts. During these early stages in embryo development, the embryos produce soluble ligands and receptors for various autocrine and paracrine factors that support the need of the growing embryo and make the endometrium receptive for it (17). The secretome, defined as proteins secreted at any given time or under certain physiological conditions, constitutes an important group of proteins that control and regulate a multitude of biological and physiological processes (18).

Secretome analysis of culture medium, in which the embryo grows, represents a non-invasive method of retrieving information about the quality of the embryo, which together with morphology and time-lapse data could aid in the decision concerning embryo selection for transfer.

In order to develop and improve the prediction models for IVF treatment, we analysed the secretome of individual human embryos that developed into blastocysts, compared with that from arrested embryos, using Proseek Multiplex Assays (Olink Bioscience, Sweden). We also investigated if the different protein levels in the secretome corresponded to the times to different developmental stages of embryos that did and did not develop into blastocysts.

## Materials and methods

### Human embryo culture

Couples that had undergone IVF treatment at the Centre for Reproduction, Uppsala University Hospital or the Carl von Linné Clinic in Uppsala were asked to donate surplus cryopreserved embryos (*n* = 47) that, according to Swedish law, otherwise had to be destroyed after the maximum allowed storage time of five years. At the Centre for Reproduction and the Carl von Linné Clinic oocytes were inseminated in fertilization medium (G-IVF™ Plus, Vitrolife^®^, Sweden and Sydney IVF, Cook^®^, Australia, respectively) and transferred to cleavage medium (G1™ Plus, Vitrolife^®^, Sweden and Sydney IVF, Cook^®^, Australia, respectively) after evaluation of fertilization (19). Only zygotes with two pro-nuclei were selected for further culture. The embryos were further cultured in a humidified incubator at 37 °C and with 6% CO_2_/6% O_2_. On day 2, only embryos with four blastomeres, <20% fragmentation, and without abnormalities were cryopreserved at the Centre for Reproduction. Embryo scoring was routinely performed by two embryologists. At the Carl von Linné Clinic only embryos with four to six blastomeres, <20% fragmentation, a visible single nucleus in at least 50% of the blastomeres, and without major variation in blastomere size were cryopreserved on day 2. At both sites, embryos were scored 42–44 hours after insemination and cryopreserved (Cryopreservation Kit; Sydney IVF, Cook^®^, Australia).

Embryo culture for the study experiments was performed at the Centre for Reproduction, Uppsala University Hospital. A total of 47 embryos were used in four individual experiments.

Human day-2 cryopreserved embryos were thawed (Sydney IVF Thawing kit, Cook^®^, Australia), and viable embryos were transferred to an EmbryoScope^®^ (Unisense FertiliTech, Aarhus, Denmark), where they were cultured for four days (i.e. until day 6 after fertilization) in equilibrated cleavage-stage medium containing human serum albumin, insulin, salts, amino acids, and penicillin G (CCM™, Vitrolife^®^, Sweden) in 6% CO_2_ and 6% O_2_ at 37 °C. As controls to detect background levels of proteins in the culture media, culture media without embryos were used. Embryo quality was evaluated retrospectively using standard morphological criteria for cleavage-stage embryos, according to the system developed by Gardner et al. (20) and Hardarson et al. (21) using time-lapse photography generated by the EmbryoScope^®^. Scoring is based on different timing to development as well as on any abnormal features such as fragmentation, multi-nuclei, or irregular cell divisions; 6 is the highest score (hatched embryo) and 1 the lowest. The trophectoderm and the inner cell mass are also scored, where A is the highest, reflecting evenly shaped cells in the trophectoderm and normal size and shape of the inner cell mass. In our study, high-quality embryos scored ≥4BB and low-quality embryos <4BB, where 4 indicates that the embryo had developed into a blastocyst where the cavity is greater than the original volume of the embryo and the *zona pellucida* is thinned. Furthermore, letter B indicates that the inner cell mass is easily discernible, with many cells loosely grouped. A trophectoderm scored as B (the second letter) reflects few cells forming a loose epithelium.

After four days of culture in the EmbryoScope^®^ the culture medium was collected (20 μL). All samples were stored at –70 °C until further analysis.

### Multiplex proximity extension assay

The relative levels of proteins secreted into embryo culture medium were analysed by using Proseek Multiplex Assays (Olink Bioscience, Sweden), based on proximity extension assay (PEA) technology (22,23). The assay has sensitivity down to fg/mL and detects relative protein values in 1 μL samples that can be used for comparison between groups, but not for absolute quantification. The PEA technique with combined double antibodies and subsequent qRT-PCR results in high specificity and can be multiplexed without introducing cross-reactivity (22,23). For this study the Multiplex Oncology I v2^96 × 96^ panel was chosen, as many of the tumour-associated proteins are of importance for normal biological processes such as angiogenesis, cell–cell signalling, growth control, and inflammation. For quantification in the PEA, the Fluidigm BioMark™ HD real-time PCR platform was used (23). Each plate was run with three negative assay controls (buffer) and three inter-plate assay controls. Every sample was also spiked with two incubation controls (green fluorescent protein and phycoerythrin), one extension control, and one detection control.

The platform provides normalized protein expression (NPX) data on a Log_2_ scale, where a high protein value corresponds to a high protein concentration. For each protein, a limit of detection was calculated (mean for the negative control + three standard deviations), and measurements below this limit were removed from further analyses. For a protein to be detected, the limit was set to >0.3 Cq above limit of detection (LOD).

The relative levels of the 92 human proteins were measured in 47 samples of embryo culture medium and two controls (fresh culture medium and conditioned culture medium). To rule out any influence of the mineral oil covering the wells during culture of the embryos, protein levels in the mineral oil were also analysed.

### Statistical analyses

All data, including demographic data, timing to development, and protein levels in the culture media, were not normally distributed, and the Mann–Whitney *U* test was used for statistical comparisons. The data are presented as median and range (minimum–maximum). Receiver-operating characteristic (ROC) curves were constructed to assess predictive ability concerning the development of blastocysts at arbitrarily chosen time points in connection with morula formation. Sensitivity and specificity were calculated at the optimal cut-off time point chosen. Correlation between protein levels and time to morula formation was calculated using Spearman’s rank correlation analysis, where the obtained coefficient is a measurement of the strength of the correlation between protein levels and time to morula formation. A *P* value of <0.05 was considered statistically significant. All statistical analyses were performed by using the Statistical Package for the Social Sciences v. 20.0 (IBM-SPSS Inc., Chicago, IL, USA) for Windows software package.

### Ethics approval and research performance

The study was approved by the Regional Ethics Review Board, Uppsala, Sweden (2008/689-32; 2012/328). Informed consent was obtained from each couple who donated embryos for the study. All research methods were performed according to relevant guidelines and regulations.

## Results

### Human embryo development

Among the embryos used for culture, 63.8% (*n* = 30) developed into blastocysts, and the remaining 36.2% (*n* = 17) were considered arrested. The majority of blastocysts, 76.7% (*n* = 23) were high-quality blastocysts (≥4BB), whereas 23.3% (*n* = 7) were of low quality (<4BB). As shown in Table I, a significant difference in the percentage of viable cells after thawing was noted between embryos that developed into blastocysts and those that did not (i.e. were arrested) (*P* = 0.001), and there was also a significant difference when high- and low-quality blastocysts were compared (Table II) (*P* = 0.005).

**Table II.** Demographic data of embryos developing into low- or high-quality blastocysts (*n* = 30).

	Low-quality blastocysts (*n* = 7)	High-quality blastocysts (*n* = 23)	*P* value
Number of cells at start, median (range)	4 (4–7)	4 (3–8)	0.277
Number of viable cells at start, median (range)	4 (3–5)	4 (3–8)	0.900
Percentage viable cells at start, median (range)	83.33 (71–100)	100 (75–100)**	0.005

** *P* < 0.01 compared with low-quality blastocysts, Mann–Whitney *U* test.

### Timing of development

The times to different developmental stages after thawing of the embryos were retrieved from the EmbryoScope^®^. When comparing embryos that developed into blastocysts with those that were arrested, significantly shorter times to formation of a morula (51.4 h versus 61.6 h; *P* = 0.008) (Table III) and start of cavitation (63.0 h versus 83.9 h; *P* = 0.001) (Table III) were observed. Also, embryos developing into high-quality blastocysts displayed a significantly shorter time to the start of cavitation compared with those that developed into low-quality blastocysts (61.1 h versus 69.7 h; *P* = 0.039) (Table IV), and there was a tendency towards a shorter time to formation of a morula (50.6 h versus 57.2 h; *P* = 0.059) (Table IV).

**Table III.** Demographic data showing the timing to development in embryos developing into blastocysts or that arrested. Time was counted from thawing of the embryos.

	Arrested embryos *n* = 17		Blastocysts *n* = 30		*P* value
	*n*	median h (range)	*n*	median h (range)	
Time to first cleavage	16	18.0 (3.1–36.9)	30	11.8 (3.1–35.4)^b^	0.067
Time to morula	10	61.6 (46.4–78.9)	29	51.4 (31.3–72.1)**	0.008
Time to start of cavitation	5	83.9 (75.3–94.2)	30	63.0 (42.4–79.4)***	0.001
Time to start of expansion	2	88.3 (86.6–90.0)	30	71.2 (47.9–88.1)^a^	0.029
Time to start of hatching			8	87.2 (70.6–94.1)	N/A

** *P* < 0.01 compared with arrested embryos, Mann–Whitney *U* test.

*** *P* < 0.001 compared with arrested embryos, Mann–Whitney *U* test.

a Owing to the low number of arrested embryos that started to expand, the significant difference in time to this stage is not noted.

b Tendency.

**Table IV.** Demographic data showing the timing to development in embryos developing into low- or high-quality blastocysts. Time was counted from thawing of the embryos (*n* = 30).

	Low-quality blastocysts *n* = 7		High-quality blastocysts *n* = 23		*P* value
	*n*	median h (range)	*n*	median h (range)	
Time to first cleavage	7	9.3 (3.1–24.6)	23	13.1 (4.3–35.4)	0.477
Time to morula	6	57.2 (45.9–67.3)	23	50.6 (31.3–72.1)^a^	0.059
Time to start of cavitation	7	69.7 (56.6–79.3)	23	61.1 (42.4–79.4)*	0.039
Time to start of expansion	7	79.0 (60.9–88.1)	23	66.9 (47.9–87.4)	0.091
Time to start of hatching			8	87.2 (70.6–94.1)	N/A

* *P* < 0.05 compared with low-quality blastocysts, Mann–Whitney *U* test.

a Tendency.

### Secretomics of developing embryos

**Figure 1.**
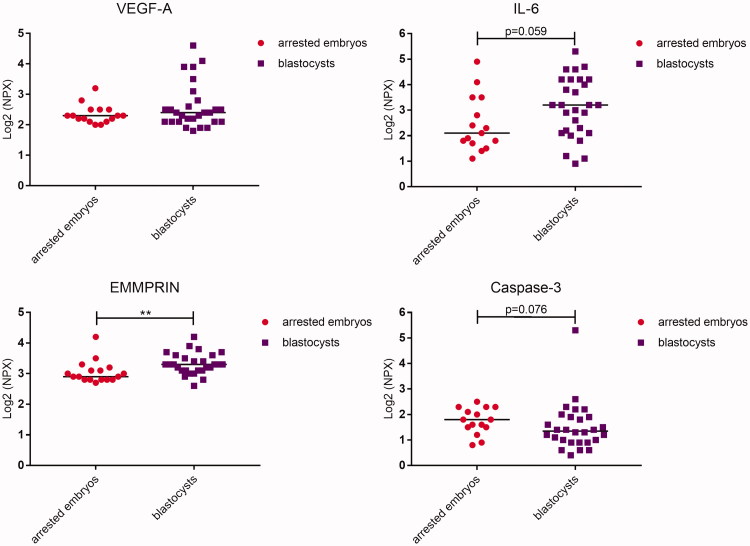
Dot plot showing median protein levels detected in culture media from human embryos developing into blastocysts or that arrested. Each dot represents the level of the respective protein from each embryo. Comparison between the groups was determined by using the Mann–Whitney *U* test. NPX = normalized protein expression.

**Figure 2.**
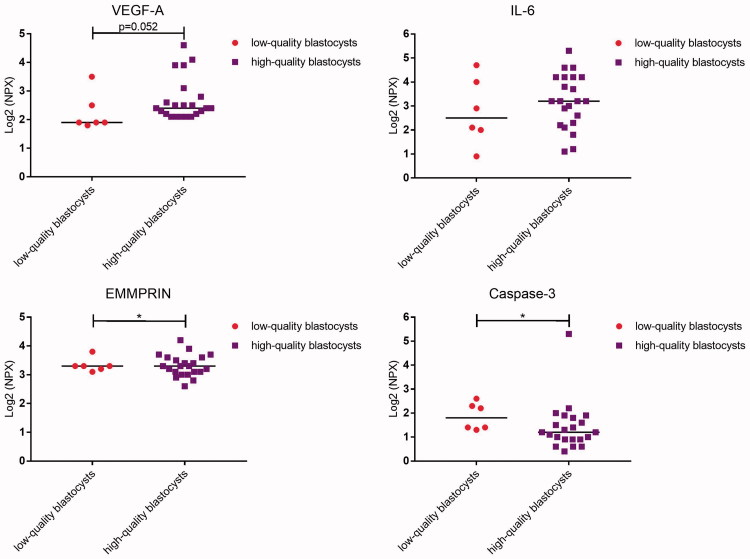
Dot plot showing median protein levels detected in culture media from human embryos developing into low-quality or high-quality blastocysts. Each dot represents the level of the respective protein from each embryo. Comparison between the groups was determined by using the Mann–Whitney *U* test. NPX = normalized protein expression.

In culture medium collected from human embryos on developmental day 6, nine proteins were detected that were at a higher concentration than background levels in conditioned medium, i.e. culture medium without embryo (Figure 1; Table V). The proteins were vascular endothelial growth factor A (VEGF-A), interleukin-6 (IL-6), extracellular matrix metalloproteinase inducer (EMMPRIN), placental growth factor (PlGF), cystatin B, epithelial cell adhesion molecule (EpCAM), caspase-3, epididymal secretory protein (HE-4), and interleukin-8 (IL-8). The levels of EMMPRIN were significantly higher in connection with embryos developing into blastocysts versus arrested embryos (*P* = 0.003; Figure 1; Table V). There was also a tendency towards higher levels of IL-6 and lower levels of caspase-3 in connection with blastocysts versus arrested embryos (*P* = 0.059 and 0.076, respectively). In high-quality blastocyst culture media, the levels of caspase-3 differed significantly compared with those in culture media from low-quality blastocysts (*P* = 0.043; Figure 2; Table VI). Furthermore, a tendency towards higher levels of VEGF-A (*P* = 0.052) was noted in culture media from blastocysts of high quality versus blastocysts of low quality.

**Table V.** Proteins detected in culture media from embryos developing into blastocysts or that arrested. The median is based on normalized protein expression.

	Arrested embryos *n* = 17			Blastocysts *n* = 30		*P* value	Background CCM™ medium	
	*n*	median (range)	*n*		median (range)		conditioned medium	fresh medium
VEGF-A	17	2.3 (2.0–3.2)	28		2.4 (1.8–4.6)	0.472	2.1 (2.1–2.1)	2.1 (2.0–2.2)
IL-6	15	2.1 (1.1–4.9)	28		3.2 (0.9–5.3)^a^	0.059	n.d.	n.d.
EMMPRIN	17	2.9 (2.7–4.2)	28		3.3 (2.6–4.2)**	0.003	2.3 (2.3–2.3)	2.4 (2.4– 2.4)
PlGF	17	0.8 (0.5–2.7)	27		0.8 (0.5–4.6)	0.274	0.6 (0.6–0.7)	0.6 (0.5–0.6)
Cystatin B	17	1.4 (0.8–2.2)	27		1.3 (0.8–3.5)	0.752	1.0 (1.0–1.1)	0.9 (0.8–1.0)
EpCAM	17	3.5 (2.6–5.5)	28		3.6 (2.7–7.2)	0.489	2.9 (2.9–2.9)	2.8 (2.8–2.9)
Caspase-3	15	1.8 (0.8–2.5)	28		1.4 (0.4–5.3)^a^	0.076	n.d.	n.d.
HE-4	16	0.7 (0.4–1.2)	28		0.7 (0.4–1.0)	0.980	n.d.	n.d.
IL-8	16	0.9 (0.6–3.7)	24		(0.5–6.1)	0.978	0.8 (0.8 – 0.8)	n.d.

** *P* < 0.01 compared with arrested embryos, Mann–Whitney *U* test.

a Tendency.

n.d. = not detected.

**Table VI.** Proteins detected in culture media from embryos developing into low- or high-quality blastocysts (*n* = 30). The median is based on normalized protein expression.

	Low-quality blastocysts *n* = 7		High-quality blastocysts *n* = 23		*P* value	Background CCM™ medium	
	*n*	median (range)	*n*	median (range)		conditioned medium	fresh medium
VEGF-A	6	1.9 (1.8–3.5)	22	2.4 (2.1–4.6)^a^	0.052	2.1 (2.1–2.1)	2.1 (2.0–2.2)
IL-6	6	2.5 (0.9–4.7)	22	3.2 (1.1–5.3)	0.369	n.d.	n.d.
EMMPRIN	6	3.3 (3.1–3.8)	22	3.3 (2.6–4.2)	0.822	2.3 (2.3–2.3)	2.4 (2.4–2.4)
PlGF	5	0.7 (0.5–2.3)	22	0.8 (0.6–4.6)	0.413	0.6 (0.6–0.7)	0.6 (0.5–0.6)
Cystatin B	6	1.2 (0.8–2.0)	21	1.3 (1.0–3.5)	0.597	1.0 (1.0–1.1)	0.9 (0.8–1.0)
EpCAM	6	3.4 (2.7–4.6)	22	3.7 (2.7–7.2)	0.261	2.9 (2.9–2.9)	2.8 (2.8–2.9)
Caspase-3	6	1.8 (1.3–2.6)	22	1.2 (0.4–5.3)*	0.043	n.d.	n.d.
HE-4	6	0.6 (0.4–0.9)	22	0.7 (0.4–1.0)	0.495	n.d.	n.d.
IL-8	3	(1.0–1.7)	21	(0.5–6.1)	0.123	0.8 (0.8–0.8)	n.d.

* *P* < 0.05 compared with low-quality blastocysts, Mann–Whitney *U* test.

a Tendency.

n.d.: not detected.

### Time to morula formation and protein levels

**Figure 3.**
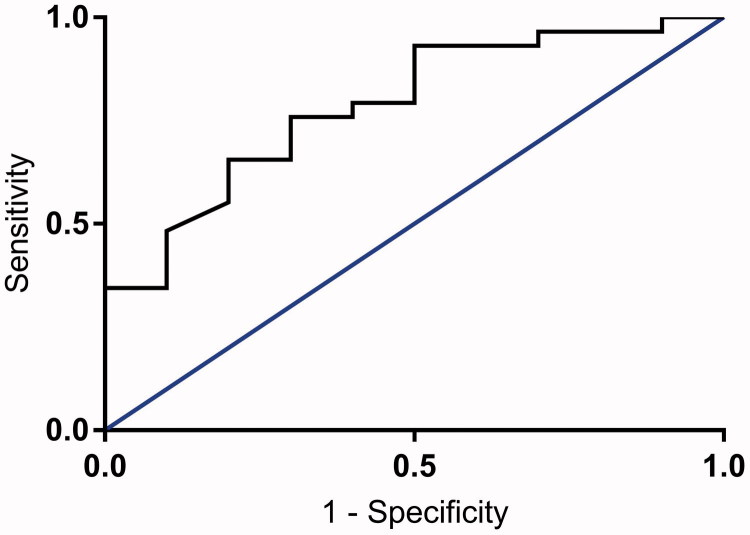
Receiver-operating characteristic curve for time to morula formation, based on whether or not the embryos developed into blastocysts. Area under curve = 0.786.

Based on the finding that a shorter time to morula formation is associated with development of blastocysts (Table III), receiver-operating characteristic (ROC) curves were constructed regarding the prediction of blastocyst development at arbitrarily chosen time points, and a cut-off value was defined (Figure 3). At a value of 51.2 h to morula formation, a sensitivity level of 48% and specificity of 90% to predict development of a blastocyst with an accuracy of 79% was found.

**Figure 4.**
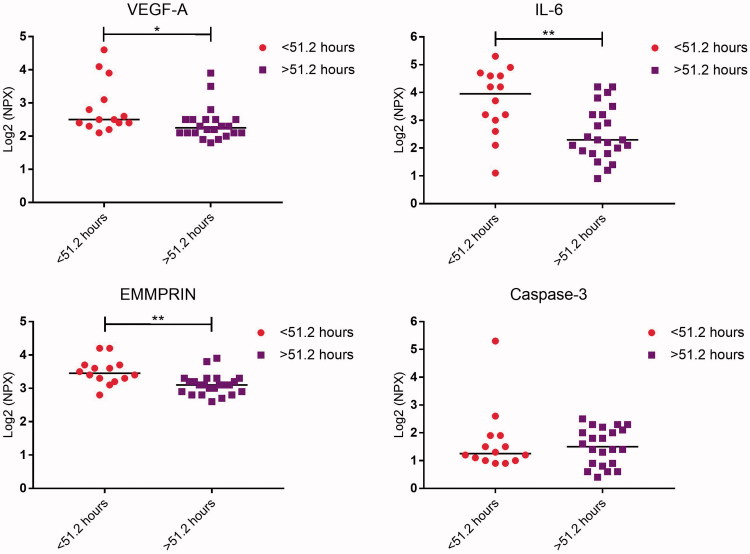
Dot plot showing median protein levels detected in culture media from human embryos developing into morulae faster or slower than the cut-off of 51.2 hours. Each dot represents the level of the respective protein from each embryo. Comparison between the groups was determined by using the Mann–Whitney *U* test. NPX = normalized protein expression.

Two groups were defined using the cut-off time for morula formation. The secretome was analysed, focusing on the proteins that differed between embryos developing into blastocysts and those that did not, as well as between high-quality and low-quality blastocysts (Tables V and VI). The levels of VEGF-A, IL-6, and EMMPRIN were significantly higher in connection with embryos with a shorter time to morula formation compared with those that developed more slowly (*P* = 0.016, 0.004, and 0.002, respectively; Figure 4; Table VII). The levels of caspase-3 were also investigated, since differences had been detected between high-quality and low-quality blastocysts, but no significant differences were found between embryos with a shorter time to morula formation compared with those that developed more slowly (*P* = 0.858; Figure 4; Table VII).

**Table VII.** Proteins detected in culture media from embryos developing faster or slower than 51.2 hours to morula formation. The median is based on normalized protein expression.

	Faster than 51.2 hours		Slower than 51.2 hours		*P* value	Background CCM™ medium	
	*n*	median (range)	*n*	median (range)		conditioned medium	fresh medium
VEGF-A	14	2.5 (2.1–4.6)	22	2.2 (1.8–3.9)*	0.016	2.1 (2.1–2.1)	2.1 (2.0–2.2)
IL-6	14	4.0 (1.1–5.3)	23	2.3 (0.9–4.2)**	0.004	n.d.	n.d.
EMMPRIN	14	3.4 (2.8–4.2)	23	3.1 (2.6–3.9)**	0.002	2.3 (2.3–2.3)	2.4 (2.4– 2.4)
Caspase-3	14	1.2 (0.9–5.3)	22	1.5 (0.4–2.5)	0.858	n.d.	n.d.

* *P* < 0.05 compared with embryos developing faster to morula, Mann–Whitney *U* test.

** *P* < 0.01 compared with embryos developing faster to morula, Mann–Whitney *U* test.

**Figure 5.**
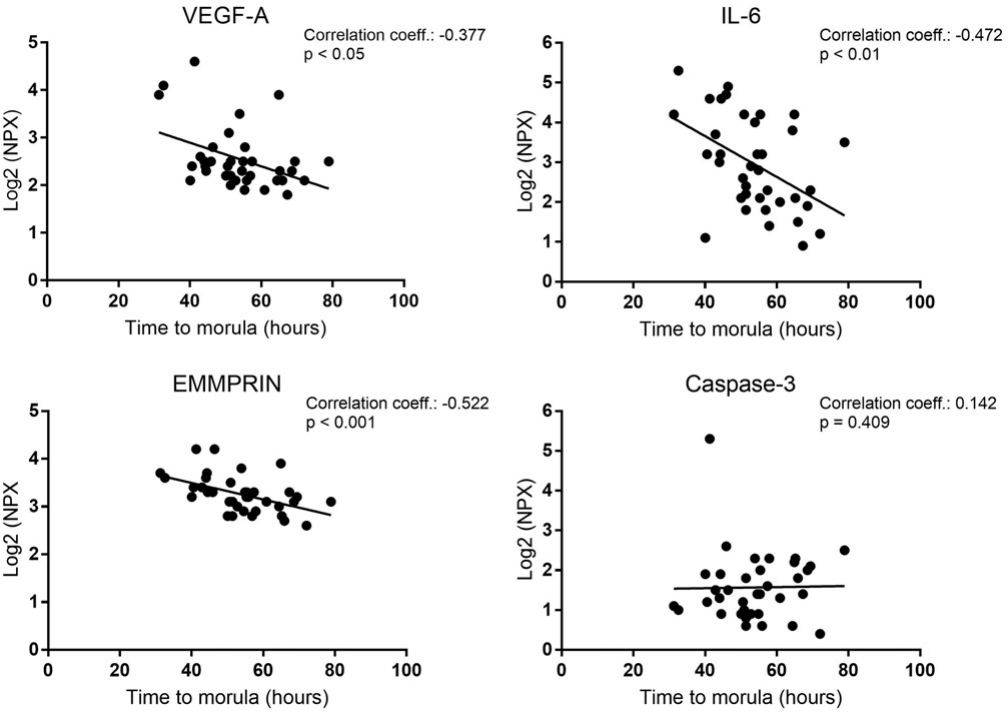
Scatter plot showing the correlation between detected protein levels and time to morula formation in human embryos. The correlation coefficient and level of significance were calculated by using Spearman’s rank correlation. NPX = normalized protein expression.

A correlation analysis was performed concerning protein levels in the media and time to morula formation (Figure 5). Significantly higher levels of VEGF-A (*P* = 0.023), IL-6 (*P* = 0.003), and EMMPRIN (*P* = 0.001) were detected in connection with embryos developing more quickly to morula stage compared with those that developed more slowly (Figure 5).

## Discussion

Human embryo development and maturation have to occur in a chronological order following certain critical steps. Timing of development and protein expression patterns might theoretically be used for prediction of viability of human embryos in connection with IVF. In our study, specific patterns of timing of development of blastocysts were found, where shorter time to morula stage correlated with the development of blastocysts, a result which is supported by those of other studies (24). Furthermore, we found that in culture medium there is a correlation between the levels of specific proteins and the formation of morulae and blastocysts. Among the detected proteins, VEGF-A, IL-6, and EMMPRIN (also known as CD147 or basigin) have been reported before as biomarkers of embryo development (17, 25–27), whereas, to our knowledge, this is the first time that PlGF, cystatin B, EpCAM, caspase-3, HE-4, and IL-8 have been detected in the secretome from early embryos and blastocysts. Interestingly, when comparing blastocysts and arrested embryos, the relative protein levels of EMMPRIN differed significantly. Furthermore, levels of IL-6 and caspase-3 in culture media tended to differ between blastocysts and arrested embryos. When analysing blastocysts only, levels of caspase-3 were significantly lower, and those of VEGF-A tended to be higher in high-quality blastocysts compared with low-quality ones, which might suggest that these proteins could be considered as potential markers of the development of high-quality blastocysts.

EMMPRIN is a multifunctional transmembrane glycoprotein receptor with various binding partners (28). A soluble form of EMMPRIN, which can be cleaved from the full-length protein in the presence of matrix metalloproteinases (MMPs), has been described (29). In agreement with our results, transcripts for EMMPRIN have been detected in human blastocysts (27), being secreted at a higher level in embryos that reached the blastocyst stage, and it has also been shown to be highly up-regulated in murine blastocysts (30). EMMPRIN is of importance in early pregnancy for adequate implantation, invasion, and differentiation of trophoblasts (31), and in most EMMPRIN-null mice the embryos fail to implant (32). Further, the protein is implicated in tumour biology, where it can increase migration, proliferation, and angiogenesis by enhancing the expression of MMPs and VEGF-A, among other things (28). Expression of EMMPRIN in association with exosome release has been reported for e.g. vessel formation, where the exosomes are released from cardiomyocytes and mesenchymal cells (33). Also, exosomes are released from bovine embryos during culturing, and these exosomes are important for blastocyst formation and quality (34). To our knowledge, there is no information on whether human embryos release exosomes, but one could assume that it is the case. However, the effect of human embryo-derived exosomes and their secreted proteins has been difficult to elucidate (35). Also, there are no reports on exosome-released EMMPRIN in human embryos.

Expression of VEGF-A has been suggested to promote implantation of the embryo and its vascular development (17), and expression of receptors for VEGF-A in the endometrium has, furthermore, been shown to be crucial for successful implantation (36). Early expression of VEGF-A in human embryos has been shown in 3-cell-stage embryos as well as in blastocysts (17,25).

Caspase-3 is an effector caspase involved in apoptosis. Transcripts for caspase-3 are found in human oocytes and in preimplantation embryos at all stages (with <20% fragmentation) (37).

IL-6 is produced by the growing embryo, and blastocysts that secrete it seem to become implanted to a greater extent than blastocysts that do not (11). We found that IL-6 was secreted at a higher level in embryos that reached the blastocyst stage than in arrested embryos. IL-6 plays an important role in preimplantation development of the embryo, and there is growing evidence that it is of importance for blastocyst hatching, implantation, establishment of the placenta, and immune tolerance of the pregnancy (38, 39). The IL-6 receptor is found on blastocysts, trophoblasts, and endometrium (26).

Although not significant when comparing high- and low-quality blastocysts, expression levels of PlGF, cystatin B, EpCAM, HE-4, and IL-8 in culture media are of interest. Previous research has concerned attempts to establish the role of PlGF in embryogenesis and implantation, but PlGF has not been detected in culture medium from human embryos (11). Thus, our study is the first to show that PlGF, as a protein, is secreted from human embryos in culture, and we suggest that the reason for detection in our study is the sensitivity of the PEA method used. The finding is in line with the results of a recent study carried out to investigate the gene expression profile of trophectoderm cells from day-5 blastocysts, where PlGF expression was reported to be up-regulated (40). The receptor for PlGF, kinase insert domain receptor (KDR), was up-regulated in endometrial cells at the same time (40). These findings suggest that PlGF has an autocrine role in trophoblast function during early embryo development and also a paracrine role in the subsequent angiogenesis of implantation and placentation, since trophectoderm cells at this stage were found without expression of KDR (40, 41).

The proteins cystatin B, EpCAM, HE-4, and IL-8 are all of known relevance as regards human embryo development (42,43), HE-4 being a protease inhibitor involved in sperm maturation (42,43). Cystatin B has a role in protecting against the proteases leaking from lysosomes. One could speculate that cystatin B might be secreted by surrounding blastomeres expressing caspase-3 in the embryo, as an attempt to inhibit the release of lysosomal proteases from dying cells. EpCAM is a transmembrane glycoprotein and is known as a surface marker on undifferentiated human embryonic stem cells, where it is of relevance for embryonic stem cell proliferation and differentiation (44,45). EpCAM up-regulates the expression of FABP5, MYC, and cyclins A and E, all proteins known to be important for numerous biological processes, e.g. fatty acid and glucose metabolism processes and cell cycle regulation. Previous investigators reported that the presence of human blastocysts up-regulates the production of IL-8 by epithelial cells of the endometrium, but they failed to detect secretion of IL-8 by arrested embryos and blastocysts (46). However, they used a much smaller sample volume and a less sensitive assay, which might explain the lack of detection. Both receptors for IL-8 (CXCR1 and CXCR2) are expressed in the human endometrium (47), suggesting a role for IL-8 in the cross-talk between blastocyst and endometrium.

The most commonly used technique for selecting embryos for transfer is based on embryo morphology (2), where timing of development is studied. This is, however, a non-standardized method and limited due to inter-individual variability, since evaluation of morphology is performed manually. In previous studies, the impact of using time-lapse photography has been shown to correlate well with increased success rates after IVF treatment (48). Timing to morula formation has been shown to be crucial for the ability of the embryo to develop into a blastocyst (24), and our results are in agreement with this. We also conclude that timing to morula formation is of major importance as regards the formation of high-quality blastocysts. We also showed that the levels of protein expression and time to morula formation were significantly correlated. The protein levels of VEGF-A, IL-6, and EMMPRIN were significantly higher in the embryos that had a shorter time to morula formation.

The detection method used for analysing proteins involved only 1 μL of culture medium. The multiplex proximity extension assay (PEA) is a unique and highly sensitive method that involves two dedicated antibodies and a qRT-PCR set-up. This technology can be multiplexed without introducing cross-reactivity (22,23). The benefit of the method is the possibility of detecting several proteins at the same time in a small volume. For this purpose, a panel of 92 known biomarker proteins was used, many of which have been found to be of relevance in embryogenesis, implantation, and early trophoblast invasion. This technology could be an alternative method to detect biomarkers in culture medium on a routine basis, and be a complement to the predictive methods used today. We have previously tried to analyse these protein levels by mass spectrometry but failed as a result of the high content of other compounds in the culture media that masked the differences in protein levels. With PEA it is possible to detect small differences in protein levels, which is of great importance when analysing the secretome from a few cells.

Finally, a number of proteins that we expected to detect were not identified. These proteins include heparin-binding EGF-like growth factor (HB-EGF), transforming growth factor alpha (TGF-alpha), tumour necrosis factor (TNF, formerly known as TNF-alpha), and C-X-C motif chemokine 13 (CXCL13) (17). The lack of detection of these proteins could have several explanations. Potentially, embryos that are arrested in development may discharge proteases that could degrade secreted proteins, making the comparison between blastocysts and arrested embryos more complicated.

One limitation of this study is that the samples were collected when ending embryo culture on day 6, meaning that the protein analysis reflects a pool of proteins secreted throughout that period of embryo development. Also, these results are true for cryopreserved embryos that have been cultured after thawing. Embryos cultured from fertilization until transfer to the woman might show other protein levels in the secretome, especially if cultured in one-step medium.

One could argue that this study is based on a small sample size. However, this study should be considered as a pilot study that needs to be repeated not only with a prospective study design, but with a larger sample size. The embryos were surplus embryos, but they were originally cryopreserved for transfer at a later stage, meaning that they were not discarded as a result of poor quality, or arrested early in development. Also, there have been reports that the culture media themselves contain undeclared proteins (49), and we did detect some of the proteins of the Multiplex Oncology I v2^96 × 96^ panel in the culture media, but the proteins that we detected in the secretome were above background levels, meaning levels detected in culture media without any embryos.

A strength of this study is that the samples were of human origin, handled and cultured according to strict routines, and not based on animal studies. In addition, the secretome was detected in the culture media and not in the embryo *per se*. In addition, differences in protein levels were detected in a very small volume of culture medium, which is promising when searching for methods to be used in routine laboratories in order to improve the decision models for embryo transfer.

In conclusion, timing to development and the secretome seem to differ between embryos forming blastocysts of high quality compared with low quality, as well as arrested embryos. The findings are of relevance for embryo selection and might, hopefully, after extended investigations, be of importance in clinical practice where a theoretical specific protein pattern in combination with timing of development might help the embryologist to choose the most optimal embryo for transfer when performing IVF.

## Figures and Tables

**Table:** Table I. Demographic data of embryos developing into blastocysts or that arrested.

	Arrested embryos (*n* = 17)	Blastocysts (*n* = 30)	*P* value
Number of cells at start, median (range)	4 (3–8)	4 (3–8)	0.573
Number of viable cells at start, median (range)	3 (1–6)	4 (3–8)**	0.002
Percentage viable cells at start, median (range)	75 (25–100)	100 (71–100)***	0.001

** *P* < 0.01 compared with arrested embryos, Mann–Whitney *U* test.

*** *P* < 0.001 compared with arrested embryos, Mann–Whitney *U* test.
